# Hearing loss, tinnitus, and hypertension: analysis of the baseline data from the Brazilian Longitudinal Study of Adult Health (ELSA-Brasil)

**DOI:** 10.6061/clinics/2021/e2370

**Published:** 2021-03-19

**Authors:** Alessandra Giannella Samelli, Itamar Souza Santos, Fernanda Yasmim Odila Maestri Miguel Padilha, Raquel Fornaziero Gomes, Renata Rodrigues Moreira, Camila Maia Rabelo, Carla Gentile Matas, Isabela M. Bensenor, Paulo A. Lotufo

**Affiliations:** I Departamento de Fisioterapia, Fonoaudiologia e Terapia Ocupacional, Faculdade de Medicina (FMUSP), Universidade de Sao Paulo, Sao Paulo, SP, BR.; II Servico de Audiologia, Hospital Universitario, Universidade de Sao Paulo, Sao Paulo, SP, BR.; III Centro de Pesquisa Clinica e Epidemiologica, Hospital Universitario, Universidade de Sao Paulo, Sao Paulo, SP, BR.; IV Departamento de Clinica Medica, Faculdade de Medicina (FMUSP) e Hospital Universitario, Universidade de Sao Paulo, Sao Paulo, SP, BR.

**Keywords:** Hearing Loss, Tinnitus, Hypertension, Elderly, Diabetes Mellitus

## Abstract

**OBJECTIVES:**

To investigate the association among hypertension, tinnitus, and sensorineural hearing loss and evaluate the influence of other covariates on this association.

**METHODS:**

Baseline data (2008-2010) from the Brazilian Longitudinal Study of Adult Health (ELSA-Brasil) were analyzed. Altogether, 900 participants were evaluated. The baseline assessment consisted of a 7-hour examination to obtain clinical and laboratory variables. Hearing was measured using pure-tone audiometry.

**RESULTS:**

Overall, 33.3% of the participants had hypertension. Participants with hypertension were more likely to be older, male, and diabetic compared to those without hypertension. The prevalence of tinnitus was higher among hypertensive participants and the odds ratio for tinnitus was higher in participants with hypertension than in those without hypertension. However, the difference was not significant after adjusting for age. Audiometric results at 250-8,000 Hz were worse in participants with hypertension than in those without hypertension in the crude analysis; however, the differences were not significant after adjustment for age, sex, diagnosis of diabetes, and exposure to noise. No significant difference was observed in hearing thresholds among participants having hypertension for <6 years, those having hypertension for ≥6 years, and individuals without hypertension.

**CONCLUSION:**

Hearing thresholds were worse in participants with hypertension. However, after adjusting for age, sex, diagnosis of diabetes, and exposure to noise, no significant differences were observed between participants with and without hypertension. A higher prevalence of tinnitus was observed in participants with hypertension compared to those without hypertension, but without significance after adjusting for age.

## INTRODUCTION

Cardiovascular risk factors such as hypertension, diabetes mellitus, and dyslipidemia have been suggested to be associated with sensorineural hearing loss (SNHL) in previous studies (1-3). Cochlear circulatory insufficiency might be an underlying mechanism leading to SNHL in the presence of cardiovascular risk factors that affect the function of the inner ear. Malfunction of the strial vasculature may decrease cochlear oxygen supplementation, disrupt ionic recycling, increase free radical production, and accelerate cell loss. Possibly, the basal portion of the cochlea, which is responsible for the higher frequencies, is particularly vulnerable to this process (1,4-6).

Similarly, it is possible that these changes in cochlear microcirculation resulting from cardiovascular risk factors may be associated with SNHL and act as supporting factors in the pathophysiology of tinnitus (7).

Age-related hearing loss affects more than 30% of the adults aged over 50 years and its prevalence roughly doubles with each decade of life (from 45% in individuals aged 60-69 years to above 80% in individuals aged above 80 years), making it the third leading chronic health condition among aging adults (8-11).

Hearing loss (HL) impairs communication and leads to social isolation. The resulting low self-esteem can cause depression, cognitive decline, and dementia (6,9,11,12). Thus, prevention of HL is an important public health target to mitigate its adverse effects. Therefore, it is necessary to identify the modifiable risk factors for HL (6).

Several studies have been conducted, especially in high-income countries, to investigate the detrimental effect of hypertension and other cardiovascular risk factors on hearing, yielding contradictory results (1,6,11,13,14). If these cardiovascular risk factors are positively associated with HL, early intervention can be beneficial for the prevention of HL and its adverse effects. Hence, it is necessary to conduct more studies on this subject, especially in low-income and middle-income countries.

Therefore, this study aimed to investigate the association among hypertension, tinnitus, and SNHL and assess the influence of other factors such as age, sex, exposure to noise, diagnosis of diabetes, and duration of hypertension by analyzing the data from the Brazilian Longitudinal Study of Adult Health (ELSA-Brasil).

## METHODS

### Study design

This ancillary cross-sectional study included 900 ELSA-Brasil participants from São Paulo (total ELSA-Brasil participants in São Paulo: 5,061) who were invited to participate in the study and agreed to undergo audiometric testing as a part of ELSA-Brasil’s baseline assessment. From the original sample (N=901), one individual was excluded due to missing data regarding antihypertensive medications, resulting in a study sample of 900 participants.

Informed consent was obtained from all participants. The study was approved by the Ethics Committee of the University Hospital of the University of São Paulo (n 883/09).

The design, objectives, and cohort profile of ELSA-Brasil have been published in detail in previous reports (15,16). It is a prospective cohort study including 15,105 civil servants from six Brazilian cities (São Paulo, Belo Horizonte, Porto Alegre, Salvador, Rio de Janeiro, and Vitória). All active or retired employees aged 35-74 years were eligible for inclusion in the study. The baseline assessment consisted of a 7-hour examination. The examinations were conducted from August 2008 to December 2010. Blood samples were obtained after overnight fasting and glucose tolerance test (75g glucose orally) and glycated hemoglobin (HbA1c) measurements were performed (17).

### Hearing examination

After otological inspection, an audiological assessment was conducted. Screening acoustic immittance measurements (Madsen Otoflex 100, Natus Medical Incorporated, CA, USA) were performed to exclude middle ear disorders. Pure-tone audiometry was performed using air conduction at octave frequencies from 250-8,000 Hz and bone conduction at 500-4,000 Hz.

Speech tests included the speech reception threshold (SRT) and speech discrimination score (SDS). SRT assesses an individual’s ability to hear and understand standardized three-syllable words (threshold in decibel hearing level [dBHL]). SDS evaluates an individual’s ability to hear and understand standardized one-syllable words (percentage of words correctly identified).

All tests were performed using a Madsen Itera II audiometer (Natus Medical Incorporated, CA, USA) in a soundproof room (18).

### Study variables

Sociodemographic characteristics and medical and occupational histories were obtained. Hypertension was defined as reported use of medications to treat hypertension, systolic blood pressure ≥140 mmHg, or diastolic blood pressure ≥90 mmHg. Diabetes was defined as medical history of diabetes, reported use of medications to treat diabetes, fasting serum glucose ≥126 mg/dL, HbA1c level ≥6.5%, or glucose level ≥200 mg/dL at 2 hours after oral glucose tolerance test with 75g of glucose. Dyslipidemia was defined as the reported use of lipid-lowering treatment or low-density lipoprotein cholesterol level ≥130 mg/dL (17,19).

Audiometric and speech test variables were compared between individuals with and without hypertension. The mean values were calculated for audiometric frequencies (hearing threshold by frequency) in both ears and for SRT and SDS of both ears. Eventually, for individuals with absent hearing thresholds at a specific frequency at the maximum limit of the audiometer, the maximum value of the audiometer plus 1 dB was considered for the calculations. This procedure was necessary in less than 0.2% of the hearing tests. The presence of HL was defined as hearing threshold >25 dBHL at each audiometric frequency (20). In addition, the mean values for the low- to middle range frequencies (250-2,000 Hz) and those for the high-range frequencies (3000-8,000 Hz) were calculated. The tinnitus variable was also investigated, considering the individual’s perception of the symptom in any ear or head.

### Statistical analysis

Continuous variables were expressed as mean±standard deviation and median (25^th^-75^th^ percentiles) and categorical variables were expressed as proportions. The chi-squared test, Kruskal-Wallis test, and one-way analysis of variance were used as applicable. Linear regression models were built using the hearing threshold values, SRT, and SDS as dependent variables to evaluate their association with hypertension. The following models were constructed: (A) crude, (B) adjusted for age, (C) fully adjusted (adjusted for age, sex, and diagnosis of diabetes), and (D) fully adjusted, excluding individuals with a history of noise exposure. The Holm-Bonferroni correction was used to adjust *p*-values for multiple comparisons.

Similar models were built including only the hypertensive individuals with complete data on age at diagnosis (N=294) to determine if the time from the diagnosis of hypertension was associated with HL. In this analysis, the cut-off times were set at the sample median (6 years).

The odds ratio (OR) was calculated considering the number of individuals with HL in each frequency range (low-middle or high) with and without hypertension.

All analyses were performed using the R software, version 3.1.2 (The R foundation, Vienna, Austria). The significance level was set at *p*<0.05.

## RESULTS

Among the 900 participants, 300 (33.3%) had hypertension. Table 1 shows the baseline characteristics of the ELSA-Brazil study population. Participants with hypertension were older (55 *vs*. 47 years), more likely to be male (54% *vs*. 43.8%), and diabetics (31.7% *vs*. 8.5%) when compared to those without hypertension. Glucose, HbA1c, systolic and diastolic blood pressure, triglycerides, and creatinine levels were significantly higher in individuals with hypertension than in those without hypertension. Results of audiometric testing at 250-8,000 Hz and SRT were worse in participants with hypertension than in those without hypertension. The history of exposure to noise was similar in both the groups (approximately 39%). Notably, the prevalence of tinnitus was higher among hypertensive individuals (45.8% *vs*. 39.2%) (Table 1). The point estimate OR suggested a positive association between tinnitus and hypertension, but this relationship was not statistically significant after adjusting for age (OR=1.21, confidence interval: 0.90-1.62, *p*=0.197).

The audiometric measurements were also analyzed using linear models. Table 2 shows the beta-coefficients for the association between audiometric measurements and diagnosis of hypertension. In the crude model, most of the audiometric measurements were significantly worse in participants with hypertension than in those without hypertension. However, the differences were mostly non-significant when the models were adjusted for age, sex, diagnosis of diabetes, and exposure to noise. Two (non-adjusted) significant *p*-values were observed for the association between the frequencies of 6 and 8 kHz in the left ear and hypertension. However, after adjustment for multiple comparisons, both associations were no longer significant (*p*=0.510 and *p*=0.176, respectively).

A subgroup analysis of hypertensive participants was also performed to evaluate the association between HL and the time from the diagnosis of hypertension, comparing these individuals with those without hypertension (Table 3).

**Figure 1:**
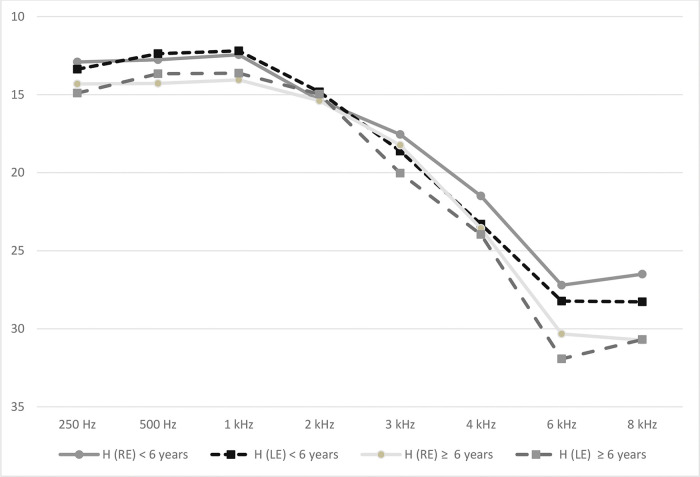
Means of hearing thresholds in the subgroup with time from the diagnosis of hypertension <6 years (N=141) and in the subgroup with time from the diagnosis of hypertension ≥6 years (N=153). RE, right ear; LE, left ear; H, hypertension.

There were no significant differences in the hearing thresholds after adjustment for age or in fully adjusted models (Table 3) between individuals diagnosed with hypertension <6 years before the data collection and individuals without hypertension. For individuals having hypertension for ≥6 years (time from diagnosis ≥6 years), statistically significant associations were observed between hypertension and hearing thresholds at 2 kHz and 8 kHz in the left ear in fully adjusted models. However, these associations were not significant after correction for multiple comparisons (*p*=1.000 and *p*=0.096, respectively). The representation of hearing thresholds by frequency for each ear of hypertensive individuals divided by the time from the diagnosis of hypertension is depicted in Figure 1.

Table 4 shows the number of individuals with HL in each frequency range (low-middle and high). The OR for high frequency range showed a difference between individuals with and without hypertension (crude model). However, the difference disappeared in the adjusted model.

## DISCUSSION

Based on the baseline data from ELSA-Brasil, the association between hypertension and SNHL was investigated in 900 ELSA-Brasil participants from the São Paulo investigation center. In the adjusted analyses controlled for multiple risk factors, there was no association between hypertension and HL or tinnitus.

Significant differences were observed between individuals with and without hypertension in demographic characteristics (age and sex) and in other risk factors (diabetes, glucose, HbA1c, dyslipidemia, triglycerides, and creatinine).

Altogether, 33% of the individuals from our sample had hypertension. Participants with hypertension were more likely to be older and male when compared with individuals without hypertension, which is consistent with the trend observed in previous studies (1,5,8,21) as well as with the characteristics of ELSA-Brasil participants at baseline (22).

Notably, the prevalence of tinnitus was higher in participants with hypertension than in those without hypertension (45.8% *vs*. 39.2%) and the OR for tinnitus was higher in hypertensive participants than in those without hypertension, although the difference was not significant after adjusting for age. A recent systematic review on hypertension and tinnitus (7) concluded that there is an association between tinnitus and hypertension, although the relationship between the cause and the effect is uncertain. The authors also stated that changes in the cochlear microcirculation caused by hypertension might be supporting factors in the pathophysiology of tinnitus. In the aforementioned review, the authors found five studies that assessed the prevalence of tinnitus in patients with hypertension and the prevalence ranged from 7.8 to 52%.

A cross-sectional observational study by the same authors (23) found that the prevalence of hypertension in individuals with tinnitus was 44% compared to individuals without tinnitus (31.4%). The study emphasized the association between tinnitus and hypertension, especially in older individuals. It is worth mentioning that the prevalence of hypertension and tinnitus increases with age and the variation observed among different studies is influenced by the study population. Our findings suggest that there is no independent association between these variables.

In the crude model, pure-tone audiometry thresholds and speech test results were significantly worse in participants with hypertension than in those without hypertension. These findings are consistent with the findings from previous studies showing that individuals with hypertension were at a high risk of HL (8,24-28). However, except those at the frequencies of 6 and 8 kHz in the left ear, the hearing thresholds did not show significant differences between individuals with and without hypertension after adjusting for age. This analysis suggested that the association between hypertension and SNHL was mostly due to the confounding effect of age. Previous studies have shown the influence of age on hearing thresholds (8,9,21,29,30). Indeed, age is the most important risk factor for HL and the audiological configuration of presbycusis has the same audiometric characteristics as the characteristics of HL due to hypertension (bilateral and symmetrical SNHL at high frequencies) (1). Additionally, when other putative confounding variables (sex, presence of diabetes, and noise exposure) were included in the models, the association between hypertension and hearing thresholds was non-significant.

These findings are consistent with the findings reported by Rey et al. (31), Baraldi et al. (32), Shargorodsky et al. (33), Lin et al. (9), Oron et al. (34), and Meneses-Barriviera et al. (21), who did not find a positive association between hypertension and HL. Similarly, Reed et al. (6) failed to establish a relationship between hypertension and HL in elderly individuals in a cross-sectional study, but found a positive association between midlife hypertension and poor hearing measured 25 years later.

In contrast, some studies have found an increased risk of HL in individuals with hypertension (2,13,35). Lin et al. (9) suggested that a possible explanation for these inconsistent results is that cardiovascular risk factors are only weakly associated with HL and their effects might be masked by stronger risk factors such as age, particularly in cohorts focused on older individuals.

To evaluate the association between HL and the time from the diagnosis of hypertension, analyses were performed to categorize hypertensive individuals according to the time from the diagnosis, with a cut-off at the sample median (6 years). However, the differences between these subgroups were not significant when the model was fully adjusted or after correction for multiple comparisons.

Although our results did not find a positive association between the time from the diagnosis of hypertension and HL, it is important to monitor this population to confirm the validity of this hypothesis. Bao et al. (14) investigated the effect of blood pressure variability (BPV) on hearing. Their findings suggested that a long-term increase in BPV was associated with HL. The authors also emphasized that a higher BPV was more likely to lead to an unstable blood supply to the inner ear, resulting in cell death and reduced hearing sensitivity. They concluded that lowering the BPV was a novel target for preventing HL.

We used another way of data analysis, considering the audiometric results by the frequency range. In the crude model, an increased risk of HL was observed for high-range frequencies in the hypertensive group than in the non-hypertensive group. However, after adjusting for age, sex, and diabetes, this difference disappeared. As discussed previously, the influence of confounding variables on hearing thresholds can be observed in this finding. In fact, chronic diseases as well as HL increase with age and all conditions affect the blood microcirculation of the cochlea, resulting in SNHL (5).

Other aspects that contribute to the variability of findings among various studies should be reinforced. As an explanation for some positive results found in cross-sectional studies, but not reproduced in longitudinal analyses, Shargorodsky et al. (33) suggested that this variability was partly due to the differences in study designs. Other methodological aspects directly affect the results and therefore, should be carefully analyzed. Many studies have been conducted using self-reported questionnaires, which may underestimate the true prevalence of a disorder (8). Differences in the cut-off values, classification criteria and studied populations (sex, race, and age) directly influence the results. Therefore, they should be considered in the analysis and comparison of the findings of different studies (8,9).

The present study found no association between hypertension and worse hearing thresholds after adjusting for age, sex, and the presence of diabetes. It should be noted that the diagnosis of hypertension was based on objective measures and the hearing thresholds were obtained through pure-tone audiometry, the gold standard for audiological assessment. Confounding variables such as age, sex, presence of diabetes, noise exposure, and duration of hypertension have not been examined simultaneously in previous studies on the effect of hypertension on SNHL. However, the mean age of the participants and the duration of hypertension were relatively low in our study population, which may have reduced the power of our study to detect positive associations.

## CONCLUSION

Hearing thresholds were worse in participants with hypertension. However, after adjusting for age, sex, and the presence of diabetes, no significant differences were found between participants with and without hypertension. A higher prevalence of tinnitus was observed in hypertensive participants than in those without hypertension, but the difference was not significant after adjusting for age.

## AUTHOR CONTRIBUTIONS

Samelli AG was responsible for the study conception and design, acquisition, analysis, and interpretation of the data, manuscript writing/editing and critical revision for important intellectual content, approval of the final manuscript version to be published, and agreement to be accountable for all aspects of the work, ensuring that the questions related to the accuracy or integrity of any part of the work were appropriately investigated and resolved. Santos IS was responsible for the analysis and interpretation of the data, critical revision of the manuscript for important intellectual content, approval of the final manuscript version to be published, and agreement to be accountable for all aspects of the work, ensuring that the questions related to the accuracy or integrity of any part of the work were appropriately investigated and resolved. Padilha FYOMM, Gomes RF, Moreira RR, Rabelo CM and Matas CG were responsible for the data acquisition, manuscript editing and review, approval of the final manuscript version to be published, and agreement to be accountable for all aspects of the work, ensuring that the questions related to the accuracy or integrity of any part of the work were appropriately investigated and resolved. Bensenor IM and Lotufo PA were responsible for the study conception, manuscript critical revision for important intellectual content, approval of the final manuscript version to be published, and agreement to be accountable for all aspects of the work, ensuring that the questions related to the accuracy or integrity of any part of the work were appropriately investigated and resolved.

## Figures and Tables

**Table 1:** Baseline characteristics of the study participants.

	No hypertension N=600	Hypertension N=300	Total N=900	*p*-value
Age in years (Median [P25-P75])	47.0 [43.0-55.0]	55.0 [48.0-63.0]	49.0 [44.0-58.0]	0.000
Male sex, N (%)	263 (43.8%)	162 (54.0%)	425 (47.2%)	0.005
Systolic BP (mmHg) (mean±SD)	114.4±11.7	132.8±17.6	120.5±16.4	0.000
Diastolic BP (mmHg) (mean±SD)	72.1±8.3	83.0±10.8	75.8±10.5	0.000
Diabetes, N (%)	51 (8.5%)	95 (31.7%)	146 (16.2%)	0.000
Fasting plasma glucose (mg/dl) (mean±SD)	101.0±15.2	116.6±40.2	106.2±27.3	0.000
HbA1c (%) (mean±SD)	5.2±0.6	5.7±1.2	5.3±0.9	0.000
Dyslipidemia, N (%)	240 (40.0%)	144 (48.0%)	384 (42.7%)	0.027
HDL cholesterol (mg/dl) (mean±SD)	52.8±12.7	50.0±10.7	51.9±12.1	0.001
LDL cholesterol (mg/dl) (mean±SD)	118.8±32.6	117.0±35.3	118.2±33.5	0.436
Triglycerides (mg/dl) (median [P25-P75])	100.1 [73.8-144.7]	120.7 [93.9-161.4]	108.8 [78.5-150.2]	0.000
Serum creatinine (mg/dl) (mean±SD)	0.9±0.2	1.0±0.3	0.9±0.2	0.000
Tinnitus, N (%)	235 (39.2%)	137 (45.8%)	372 (41.4%)^a^	0.069
Noise exposure, N (%)	222 (39.9%)	110 (39.3%)	332 (39.7%)^b^	0.933
250 Hz RE (dBHL) (mean±SD)	12.3±9.4	13.5±9.7	12.7±9.5	0.066
500 Hz RE (dBHL) (mean±SD)	11.6±9.6	13.5±10.0	12.3±9.8	0.007
1000 Hz RE (dBHL) (mean±SD)	12.1±10.5	13.3±11.0	12.5±10.6	0.113
2000 Hz RE (dBHL) (mean±SD)	12.5±11.6	15.4±13.2	13.4±12.3	0.001
3000 Hz RE (dBHL) (mean±SD)	14.8±14.2	17.8±15.1	15.8±14.6	0.004
4000 Hz RE (dBHL) (mean±SD)	18.5±16.4	22.5±17.4	19.8±16.8	0.001
6000 Hz RE (dBHL) (mean±SD)	24.2±17.7	28.7±20.4	25.7±18.7	0.001
8000 Hz RE (dBHL) (mean±SD)	23.1±20.1	28.6±22.1	24.9±20.9	0.000
250 Hz LE (dBHL) (mean±SD)	12.8±8.1	14.1±10.1	13.2±8.9	0.030
500 Hz LE (dBHL) (mean±SD)	11.2±8.0	13.0±10.7	11.8±9.0	0.004
1000 Hz LE (dBHL) (mean±SD)	11.0±9.4	12.9±11.1	11.6±10.0	0.006
2000 Hz LE (dBHL) (mean±SD)	12.5±12.1	14.9±13.4	13.3±12.6	0.006
3000 Hz LE (dBHL) (mean±SD)	15.5±14.6	19.3±16.0	16.8±15.2	0.000
4000 Hz LE (dBHL) (mean±SD)	18.9±16.5	23.5±18.0	20.4±17.1	0.000
6000 Hz LE (dBHL) (mean±SD)	26.0±17.9	30.0±20.3	27.3±18.8	0.003
8000 Hz LE (dBHL) (mean±SD)	25.0±20.5	29.4±22.3	26.5±21.2	0.003
SRT RE (dBHL) (mean±SD)	13.6±8.7	15.3±9.5	14.1±9.0	0.007
SRT LE (dBHL) (mean±SD)	13.4±7.5	15.7±10.2	14.2±8.6	0.000
SDS RE (%) (mean±SD)	96.3±5.4	95.7±4.8	96.1±5.2	0.166
SDS LE (%) (mean±SD)	96.1±5.4	95.0±7.5	95.7±6.2	0.014

a Two individuals were excluded due to missing data regarding tinnitus.

b Sixty-three individuals were excluded due to missing data regarding exposure to noise.

SD, standard deviation; BP, blood pressure; HDL, high-density lipoprotein; LDL, low-density lipoprotein; RE, right ear; LE, left ear; SRT, speech reception threshold; SDS, speech discrimination score, HbA1c: glycated hemoglobin, dBHL: decibel hearing level, P: percentile.

**Table 2:** Beta-coefficients for the association between mean audiometric measurements and hypertension in the crude and adjusted models.

	Crude	Adjusted for age	Fully adjusted	Fully adjusted, without exposure to noise
250 Hz RE	1.24 (-0.08 to 2.55; *p*=0.066)	-0.06 (-1.4 to 1.29; *p*=0.934)	0.01 (-1.38 to 1.4; *p*=0.987)	-0.46 (-2.34 to 1.42; *p*=0.633)
250 Hz LE	1.36 (0.13 to 2.59; *p*=0.03)	0.22 (-1.04 to 1.48; *p*=0.736)	0.29 (-1.01 to 1.59; *p*=0.664)	0.03 (-1.74 to 1.8; *p*=0.975)
500 Hz RE	1.86 (0.51 to 3.21; *p*=0.007)	0.3 (-1.07 to 1.68; *p*=0.663)	0.33 (-1.08 to 1.75; *p*=0.645)	-0.25 (-2.22 to 1.72; *p*=0.804)
500 Hz LE	1.84 (0.59 to 3.08; *p*=0.004)	0.57 (-0.7 to 1.85; *p*=0.378)	0.54 (-0.77 to 1.86; *p*=0.417)	-0.15 (-1.97 to 1.67; *p*=0.872)
1000 Hz RE	1.19 (-0.28 to 2.67; *p*=0.113)	-0.78 (-2.26 to 0.7; *p*=0.303)	-0.71 (-2.24 to 0.82; *p*=0.360)	-1.38 (-3.46 to 0.7; *p*=0.194)
1000 Hz LE	1.93 (0.55 to 3.31; *p*=0.006)	0.09 (-1.3 to 1.48; *p*=0.902)	0 (-1.43 to 1.44; *p*=0.996)	-0.29 (-2.29 to 1.72; *p*=0.78)
2000 Hz RE	2.9 (1.21 to 4.59; *p*=0.001)	-0.03 (-1.67 to 1.62; *p*=0.976)	-0.08 (-1.78 to 1.61; *p*=0.923)	-0.32 (-2.54 to 1.9; *p*=0.776)
2000 Hz LE	2.44 (0.7 to 4.17; *p*=0.006)	-0.66 (-2.35 to 1.02; *p*=0.443)	-0.98 (-2.71 to 0.75; *p*=0.267)	0.07 (-2.27 to 2.4; *p*=0.956)
3000 Hz RE	2.96 (0.95 to 4.97; *p*=0.004)	-0.75 (-2.68 to 1.19; *p*=0.451)	-1.4 (-3.36 to 0.56; *p*=0.161)	-0.07 (-2.54 to 2.4; *p*=0.958)
3000 Hz LE	3.77 (1.68 to 5.87; *p*=0)	-0.13 (-2.14 to 1.89; *p*=0.9)	-0.85 (-2.88 to 1.18; *p*=0.412)	0.23 (-2.36 to 2.81; *p*=0.863)
4000 Hz RE	3.95 (1.63 to 6.27; *p*=0.001)	−0.37 (-2.6 to 1.86; *p*=0.745)	-1.41 (-3.63 to 0.81; *p*=0.213)	0.17 (-2.55 to 2.88; *p*=0.905)
4000 Hz LE	4.61 (2.25 to 6.96; *p*=0)	0.11 (-2.14 to 2.37; *p*=0.922)	-0.81 (-3.06 to 1.43; *p*=0.478)	0.87 (-1.84 to 3.58; *p*=0.529)
6000 Hz RE	4.53 (1.95 to 7.11; *p*=0.001)	-0.81 (-3.24 to 1.61; *p*=0.511)	-1.75 (-4.21 to 0.72; *p*=0.165)	-0.6 (-3.77 to 2.58; *p*=0.713)
6000 Hz LE	4.01 (1.42 to 6.61; *p*=0.003)	-1.66 (-4.06 to 0.75; *p*=0.177)	-2.65 (-5.09 to -0.2; *p*=0.034)	-0.54 (-3.67 to 2.58; *p*=0.734)
8000 Hz RE	5.43 (2.56 to 8.31; *p*=0)	-1.4 (-4 to 1.2; *p*=0.292)	-2.26 (-4.91 to 0.4; *p*=0.096)	-0.76 (-4.29 to 2.78; *p*=0.675)
8000 Hz LE	4.38 (1.45 to 7.31; *p*=0.003)	-2.66 (-5.3 to -0.03; *p*=0.048)	-3.48 (-6.17 to -0.8; *p*=0.011)	-1.65 (-5.22 to 1.92; *p*=0.365)

Fully adjusted models were adjusted for age, sex, and the diagnosis of diabetes. Non-corrected *p*-values are presented in the table.

RE, right ear; LE, left ear.

**Table 3:** Beta-coefficients for the association between mean audiometric measurements and hypertension with time from the diagnosis <6 years (N=141) and ≥6 years (n=153) in the crude and adjusted models.

<6 years	Crude	Adjusted for age	Fully adjusted
250 Hz RE	0.61 (-1.13 to 2.35; *p*=0.493)	0.09 (-1.81 to 1.63; *p*=0.920)	0.01 (-1.73 to 1.74; *p*=0.995)
250 Hz LE	0.61 (-1.01 to 2.24; *p*=0.461)	0 (-1.61 to 1.61; *p*=0.997)	0.2 (-1.42 to 1.82; *p*=0.811)
500 Hz RE	1.13 (-0.66 to 2.92; *p*=0.217)	0.29 (-1.46 to 2.05; *p*=0.743)	0.31 (-1.46 to 2.09; *p*=0.730)
500 Hz LE	1.21 (-0.44 to 2.87; *p*=0.150)	0.53 (-1.09 to 2.16; *p*=0.520)	0.57 (-1.07 to 2.22; *p*=0.495)
1000 Hz RE	0.38 (-1.58 to 2.33; *p*=0.707)	-0.68 (-2.58 to 1.21; *p*=0.481)	-0.73 (-2.64 to 1.18; *p*=0.454)
1000 Hz LE	1.23 (-0.61 to 3.06; *p*=0.190)	0.24 (-1.54 to 2.01; *p*=0.793)	0.13 (-1.66 to 1.93; *p*=0.884)
2000 Hz RE	2.89 (0.65 to 5.13; *p*=0.012)	1.27 (-0.82 to 3.37; *p*=0.234)	1.04 (-1.07 to 3.15; *p*=0.334)
2000 Hz LE	2.36 (0.06 to 4.66; *p*=0.045)	0.65 (-1.49 to 2.8; *p*=0.550)	0.18 (-1.98 to 2.34; *p*=0.870)
3000 Hz RE	2.71 (0.05 to 5.37; *p*=0.047)	0.66 (-1.8 to 3.13; *p*=0.597)	-0.28 (-2.72 to 2.15; *p*=0.819)
3000 Hz LE	3.07 (0.29 to 5.85; *p*=0.030)	0.94 (-1.63 to 3.51; *p*=0.475)	-0.13 (-2.66 to 2.39; *p*=0.918)
4000 Hz RE	2.97 (-0.1 to 6.05; *p*=0.058)	0.61 (-2.23 to 3.46; *p*=0.673)	-0.8 (-3.57 to 1.97; *p*=0.572)
4000 Hz LE	4.42 (1.3 to 7.55; *p*=0.006)	1.93 (-0.94 to 4.81; *p*=0.188)	0.53 (-2.26 to 3.32; *p*=0.709)
6000 Hz RE	3.03 (-0.38 to 6.44; *p*=0.082)	0.13 (-2.96 to 3.22; *p*=0.936)	-1.04 (-4.11 to 2.02; *p*=0.505)
6000 Hz LE	2.24 (-1.19 to 5.67; *p*=0.201)	-0.84 (-3.91 to 2.22; *p*=0.589)	-2 (-5.04 to 1.05; *p*=0.199)
8000 Hz RE	3.37 (-0.43 to 7.17; *p*=0.082)	-0.34 (-3.65 to 2.97; *p*=0.842)	-1.39 (-4.69 to 1.91; *p*=0.410)
8000 Hz LE	3.23 (-0.65 to 7.11; *p*=0.103)	-0.64 (-3.99 to 2.72; *p*=0.710)	-1.71 (-5.05 to 1.64; *p*=0.318)

≥6 years	Crude	Adjusted for age	Fully adjusted
250 Hz RE	2.02 (0.33 to 3.7; *p*=0.019)	0.17 (-1.57 to 1.92; *p*=0.846)	0.25 (-1.58 to 2.08; *p*=0.788)
250 Hz LE	2.15 (0.57 to 3.72; *p*=0.008)	0.54 (-1.1 to 2.17; *p*=0.521)	0.51 (-1.19 to 2.22; *p*=0.556)
500 Hz RE	2.64 (0.91 to 4.38; *p*=0.003)	0.44 (-1.34 to 2.22; *p*=0.629)	0.46 (-1.4 to 2.32; *p*=0.629)
500 Hz LE	2.5 (0.9 to 4.1; *p*=0.002)	0.7 (-0.95 to 2.36; *p*=0.404)	0.6 (-1.13 to 2.33; *p*=0.495)
1000 Hz RE	1.98 (0.09 to 3.87; *p*=0.041)	-0.81 (-2.73 to 1.11; *p*=0.41)	-0.62 (-2.63 to 1.39; *p*=0.544)
1000 Hz LE	2.66 (0.88 to 4.43; *p*=0.003)	0.04 (-1.76 to 1.85; *p*=0.963)	-0.02 (-1.91 to 1.87; *p*=0.982)
2000 Hz RE	2.93 (0.76 to 5.1; *p*=0.008)	-1.35 (-3.47 to 0.78; *p*=0.215)	-1.36 (-3.58 to 0.86; *p*=0.229)
2000 Hz LE	2.5 (0.27 to 4.73; *p*=0.028)	-1.99 (-4.17 to 0.19; *p*=0.073)	-2.34 (-4.61 to -0.08; *p*=0.043)
3000 Hz RE	3.39 (0.81 to 5.97; *p*=0.01)	-2.01 (-4.51 to 0.5; *p*=0.117)	-2.53 (-5.09 to 0.03; *p*=0.053)
3000 Hz LE	4.49 (1.8 to 7.18; *p*=0.001)	-1.15 (-3.76 to 1.46; *p*=0.389)	-1.66 (-4.32 to 0.99; *p*=0.219)
4000 Hz RE	5.05 (2.07 to 8.02; *p*=0.001)	-1.19 (-4.08 to 1.7; *p*=0.421)	-2 (-4.91 to 0.91; *p*=0.178)
4000 Hz LE	5.08 (2.05 to 8.1; *p*=0.001)	-1.49 (-4.41 to 1.43; *p*=0.317)	-2.12 (-5.06 to 0.81; *p*=0.156)
6000 Hz RE	6.15 (2.85 to 9.45; *p*=0)	-1.51 (-4.65 to 1.63; *p*=0.345)	-2.36 (-5.58 to 0.86; *p*=0.151)
6000 Hz LE	5.94 (2.62 to 9.26; *p*=0)	-2.2 (-5.31 to 0.92; *p*=0.167)	-3.15 (-6.35 to 0.04; *p*=0.054)
8000 Hz RE	7.6 (3.92 to 11.28; *p*=0)	-2.19 (-5.55 to 1.17; *p*=0.202)	-2.93 (-6.39 to 0.54; *p*=0.098)
8000 Hz LE	5.65 (1.89 to 9.41; *p*=0.003)	-4.56 (-7.97 to -1.16; *p*=0.009)	-5.31 (-8.83 to -1.8; *p*=0.003)

Fully adjusted models were adjusted for age, sex, and the diagnosis of diabetes. Non-corrected *p*-values are presented in the table.

RE, right ear; LE, left ear.

**Table 4:** Number of individuals with hearing loss in each range of frequencies (low to middle or high) with and without hypertension, ORs, and *p*-values in the crude and adjusted models.

	Low- to middle-range of frequencies N (%) hearing loss	High-range of frequencies N (%) hearing loss
Hypertension (N=300)	39 (13%)	133 (44.3%)
No hypertension (N=600)	59 (9.8%)	208 (34.6%)
Crude model (OR; 95% CI)	1.39 (0.90; 2.15)	1.51 (1.13; 2.00)
p-value	0.134	0.004
Adjusted model (OR; 95% CI)	0.88 (0.55; 1.43)	1.08 (0.70; 1.68)
p-value	0.622	0.700

The adjusted model was adjusted for age, sex, and diagnosis of diabetes.

OR, odds ratio; CI, confidence interval.
